# HIV Clients as Agents for Prevention: A Social Network Solution

**DOI:** 10.1155/2012/815823

**Published:** 2012-05-15

**Authors:** Sarah Ssali, Glenn Wagner, Christopher Tumwine, Annette Nannungi, Harold Green

**Affiliations:** ^1^Department of Women and Gender Studies, Makerere University, Kampala, Uganda; ^2^Department of Health, RAND Corporation, Santa Monica, CA 90407, USA; ^3^Faculty of Social Sciences, Makerere University, Kampala, Uganda

## Abstract

HIV prevention efforts to date have not explored the potential for persons living with HIV to act as change agents for prevention behaviour in their social networks. Using egocentric social network analysis, this study examined the prevalence and social network correlates of prevention advocacy behaviours (discussing HIV in general; encouraging abstinence or condom use, HIV testing, and seeking HIV care) enacted by 39 HIV clients in Uganda. Participants engaged in each prevention advocacy behaviour with roughly 50–70% of the members in their network. The strongest determinant of engaging in prevention advocacy with more of one's network members was having a greater proportion of network members who knew one's HIV seropositive status, as this was associated with three of the four advocacy behaviours. These findings highlight the potential for PLHA to be key change agents for HIV prevention within their networks and the importance of HIV disclosure in facilitating prevention advocacy.

## 1. Introduction

In our work with persons living with HIV/AIDS (PLHA) in Uganda, we have observed that the combination of restored health and the sense of community among one's HIV-infected peers often translate into HIV clients being instilled with self-confidence and motivation to engage in HIV protective behaviours and to encourage others to do so as well. Clients talk about and demonstrate their desire to share their experiences and to advocate for HIV testing, seeking HIV medical care, and engagement in behaviours to prevent HIV transmission. Some feel so impassioned and motivated to reach out to others that they are emboldened to be public advocates for HIV prevention and for seeking HIV care, and to do so without prompting or compensation. However, data are scarce from empirical investigations of this phenomenon.

While HIV prevention interventions increasingly target the risk behaviour of HIV-infected individuals [1–3], few have tried to take advantage of the potential of PLHA to be key facilitators of secondary prevention. Peer outreach strategies for HIV prevention have been used among at-risk populations including drug users [4–6] and sex workers [7]. A process described as “intravention,” by which members of a community engage in and advocate for behavioural change with other community members, has been successfully promoted in communities and networks of drug users [6]. However, we are not aware of any programs that focus specifically on activating PLHA to be advocates for prevention. PLHA may be the most effective messengers of prevention, as they are more likely to have greater access to at-risk individuals, to be more influential to their family, friends and peers because of their relational closeness, and more credible because of their experiences living with HIV. HIV clients on antiretroviral therapy (ART) may be particularly influential with regard to protective behaviours such as seeking HIV testing and medical care, given the often visible benefits of treatment on physical health and functioning [8, 9].

Recognizing the value of PLHA as agents for prevention and positive health behaviour change allows PLHA to be viewed as a critical part of the solution, rather than the source of the HIV/AIDS problem. To fully actualize the potential for PLHA to be changing agents in their family and community, greater understanding is needed of the social context in which PLHA discuss HIV and promote protective behaviours. We define social context broadly as the set of social relationships that surround PLHA, which is more specifically described by the structure and composition of their social networks. Social networks are one vector by which information is exchanged and spreads through a community [10–12]. Most research on social networks and HIV has been with drug users in the U.S. [6, 13], and has focused on the risk aspects of networks [14], rather than the potential benefits of spreading information about and encouragement of HIV prevention.

We are unaware of any studies that have examined the prevention advocacy behaviour of PLHA in sub-Saharan Africa and how social network characteristics may be associated with engagement in HIV prevention advocacy. To begin to fill this gap, we conducted a study of the social networks of HIV clients in Uganda and the prevalence and correlates of prevention advocacy within these networks. We describe the extent to which HIV clients discuss HIV and encourage protective behaviours with members of their social network, the content and recipients of these discussions, and the individual and network characteristics that may influence whether or not PLHA engage in specific prevention advocacy behaviours.

## 2. Methods

### 2.1. Study Participants

Participants in this cross-sectional study consisted of a convenience sample of HIV-positive adult clients attending the Infectious Diseases Institute HIV clinic in Kampala, with the sample stratified by ART status and length of time in care. All adult (age 18 or older) clients were eligible to participate. Clients were referred by clinic staff to the study interviewers for consent procedures. Though no direct data on refusals were collected, the study interviewers report that very few respondents refused to participate once informed of the study. The research protocol was approved by the Institutional Review Board at Makerere University.

### 2.2. Measures

Data were collected between February and March 2009. Computer assisted, in-person, structured interviews were conducted by trained social science researchers and graduate students in the respondent's language of choice, usually English, Luganda, or Lunyankole. Participants received 10,000 Uganda Shillings (~$5 USD) after the interview to compensate for transportation costs.

Demographic (age, sex, marital status, education level, occupation) and HIV medical (date of HIV diagnosis, CD4 count, ART treatment status, and ART start date) characteristics of the respondent were collected via self-report. For those on ART, antiretroviral adherence was measured by asking the participant to report the number of antiretroviral doses missed in the last week relative to the total number of prescribed weekly doses.

Perceptions of general social support, peer community support, HIV discrimination and internalized HIV stigma were assessed with single items taken mostly from validated scales. General social support was assessed using an item from the Adult AIDS Clinical Trials Group measures [15], “I can count on my family and friends to give me the support I need.” We developed a single item to assess community peer support amongst fellow HIV clients, “I feel a sense of community when I am with other HIV clients here at the clinic.” HIV discrimination was measured using a single item from a scale developed by Berger and colleagues [16]; participants were asked to rate their level of agreement with the statement, “My friends and family stopped visiting me when they found out I was HIV positive.” Internalized HIV stigma was assessed with a single item from a scale developed by Kalichman et al. [17]; participants were asked to rate their level of agreement with the statement, “I am ashamed that I am HIV-positive.” The response format used in all of these items ranged from 1 “strongly agree” to 4 “strongly disagree”; higher scores represented higher levels of the construct. Single items, rather than complete scales, were used to limit respondent burden and because of the pilot nature of the study. The specific items that were chosen were selected because the concepts they probed were most relevant to our research questions.

To assess the respondent's social network, we used a personal, egocentric network approach, which focuses on the network of ties that surround the respondent. Established procedures for conducting personal network interviews were employed [18–20], through which information is collected from one individual (the participant, or “ego”) about their network rather than from each member in the network. The personal network approach is designed to identify the wide range of network members (“alters”) that individuals interact within their lives and to measure participants' understanding of the connections among those alters. The interview included three sections to elicit social network information. First, participants were asked to list 20 individuals with whom they have been in communication in the past 6 months (by phone, email, in person, etc.), starting with those most important to them. These individuals could include kin (immediate and extended family), friends, acquaintances, neighbors, or people in service/helping positions (e.g., health care workers, counselors). Research has demonstrated that 20 alters can reliably capture the variability in most network characteristics [21].

Secondly, to assess network structure, respondents were asked to indicate if each unique pair of alters knows each other and how often they interact. Each affirmative response corresponds to a link in a network diagram between two alters. Based on this information one can determine, for instance, who the most important individuals are, and how interconnected the network is overall. Several network structural measures were calculated. The number of network components was measured; components are groups of three or more individuals who are completely detached (not known by) from the other members in the network, which, when combined with the size of the largest component, provides information on how fragmented a network might be. The most popular contact was determined by identifying the alter who has the most number of ties to other alters; number of isolates was measured by number of network alters not connected to other alters; network density was measured by the proportion of ties that exist relative to the number of ties that could exist.

To assess alter composition, the following data were collected with regard to each network alter: age, gender, HIV status, and if HIV-positive, whether or not they are in HIV care and on ART; information about the nature of the relationship to the respondent, including type of relationship (e.g., family, friend, provider), whether the person knows the respondent's HIV status, ratings of closeness and trust, and how much the respondent talks to, seeks advice from and receives and provides support to the alter.

Lastly, we asked whether the respondent had engaged in prevention advocacy behaviours with each alter over the past 6 months, with response options of “never,” “sometimes,” or “often.” Specifically, respondents were asked how often they had discussed the following with a network alter either face-to-face or by phone: HIV in general, abstinence or condom use, getting HIV tested (for HIV-negative alters), and seeking HIV medical care (for HIV-positive alters).

### 2.3. Analysis

We entered interview responses directly in real time into Egonet, a software used to collect social network data; data were then exported into SPSS for data analysis. We used descriptive statistics to describe the characteristics of the sample and their social networks, and the frequency of prevention advocacy behaviors, and bivariate statistics (Pearson correlations; ANOVAs) to examine relationships between specific prevention advocacy behaviours and characteristics of the participants and their social networks. Note that all analyses were conducted at the level of the participant, not at the alter or dyadic level (e.g., comparing proportion of respondent's alters with a specific characteristic, rather than the proportion of all alters with a specific characteristic).

## 3. Results

### 3.1. Sample Characteristics

Thirty-nine participants were interviewed, consisting of 19 non-ART clients in care for variable amounts of time, 10 clients on ART for 6 months or less, and 10 clients on ART for 12 months or more. Mean age was 34 years (SD = 9; range: 18 to 52), the average years of formal education was 9.8 years (SD = 3.1), and a large proportion (67%) were earning an income of some sort. The majority of the respondents were women (67%), nearly half the sample was married (49%), and another 31% were either divorced or had a spouse who had died. Mean self-reported CD4 count was 332 (SD = 174), and mean length of time since HIV diagnosis was 48 months (SD = 53; range: 2 months to 18.5 years). With respect to network composition, network members were an average of 34 years old, half were female (52%), and most were either family members (including spouses) (45%) or friends (33%). Forty-nine percent of respondents reported that at least one network alter knew the respondent's HIV status. More details on the composition of the networks are reported elsewhere [22].

### 3.2. Prevalence of Prevention Advocacy Behaviours

All participants (100%) had discussed HIV in general, and abstinence or condom use, with at least one alter, HIV testing with at least one HIV-negative alter, and seeking HIV medical care with at least one HIV-positive alter, in the past six months. Respondents reported discussing HIV with an average of 60% of all alters, abstinence and condom use with an average of 48% of alters, HIV testing with an average of 47% of alters who were thought to be HIV-negative, and seeking HIV care with 73% of alters who were known to be HIV-positive. With all participants reporting these prevention advocacy behaviours with at least one relevant alter, further analyses focused on the proportion of alters with whom these behaviours were discussed.  Table 1 lists the participant and network variables related to the proportion of alters with whom these four prevention advocacy behaviours were discussed.

### 3.3. Correlates of Prevention Advocacy Behaviours

#### 3.3.1. Discuss HIV in General

The participant characteristics significantly associated with discussing HIV were ART adherence and time since HIV diagnosis. Greater adherence was related to discussing HIV with a higher proportion of alters (*r* = 0.55, *P* < 0.01). Length of time since diagnosis was positively correlated with the proportion of alters with whom HIV was discussed (*r* = 0.44, *P* < 0.01). Older age and being on ART were marginally associated with discussion of HIV with more alters (*P* values <0.10). With regard to network composition, greater percentage of alters who know the respondent's HIV status was significantly correlated with discussion of HIV (*r* = 0.60, *P* < 0.001), while greater percentage of HIV positive members in the network was related at a trend level of significance (*P* < 0.10). The network structure variable associated with discussing HIV was the number of connections the most popular network member had to others in the network (*r* = 0.35, *P* < 0.05); network density was associated at trend levels of significance (*P* values <0.10). Stigma, social support, discrimination and peer community support were not associated with prevalence of general HIV discussions with alters.

**Figure 1:**
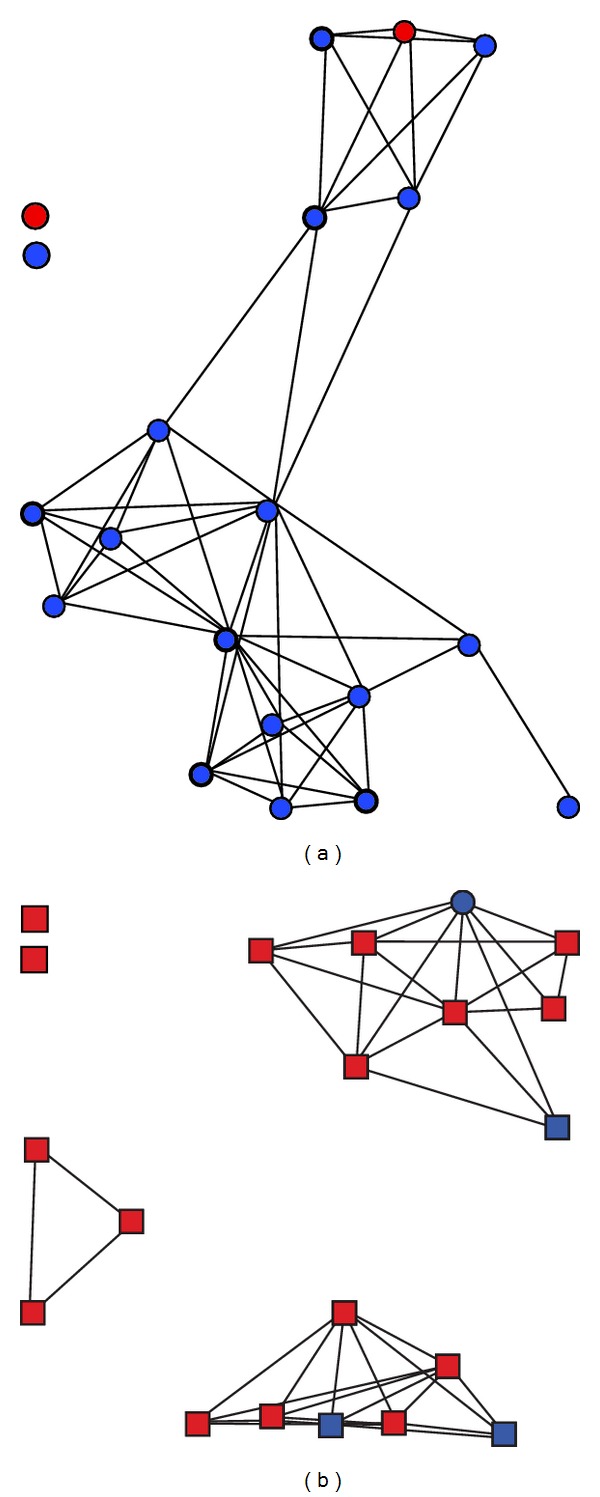
Graphical example of the relationship between social network characteristics and discussion of HIV with network members.

Figure 1 depicts network diagrams that illustrate the relationship between length of time since diagnosis, network density, alter knowledge of respondent's HIV status, and discussing HIV with network members. Respondent “a” has known their HIV-positive status for 13 years, while respondent “b” has known for less than 3 months. The twenty nodes (circles and squares in the diagrams) represent the 20 network members named by each respondent. If the respondent believed that two network members interacted often (compared to never or sometimes), there is a line between the two nodes representing those alters. Circular nodes know the respondent's HIV status. Blue nodes represent alters with whom the respondent discussed HIV. From this diagram, respondent “a,” who has known their HIV status the longest, had a more integrated social network, in which almost every member had interacted with another in the past six months, compared to respondent “b” where the network was grouped into three distinct components. Also, “a” had discussed HIV with almost everyone in the network, while “b” had discussed with only four members of the network. Furthermore, whereas everyone in the network of “a” knew the respondent's HIV status, in the network of “b,” only one person in the network knew their status.

#### 3.3.2. Discuss Abstinence or Using Condoms

The only characteristic significantly associated with discussing abstinence and condom use was the network characteristic of the percentage of alters who knew the respondent's HIV status. That is, the respondent was more likely to discuss abstinence and condom use with network members if more of the network members were perceived to know his/her HIV status (*r* = 0.35, *P* < 0.05). Time since HIV diagnosis was marginally associated with discussing abstinence or condom use (*P* < 0.10). No respondent characteristics or network structure characteristics were found to be significantly associated with discussing abstinence or condom use.

#### 3.3.3. Discuss HIV Testing among HIV-Negative Alters

Time since HIV diagnosis was marginally associated with discussing HIV testing with HIV negative alters (*P* < 0.10). No other personal characteristics were found to be significantly associated with discussing HIV testing with HIV-negative alters. Greater percentage of network members who know the respondent's HIV status was associated with the respondent discussing HIV testing with a higher proportion of network members who were HIV negative (*r* = 0.41, *P* < 0.01). With regard to network structure, the number of connections the most popular network member had with other network members, and network density were found to be associated at a trend level of significance (*P* values <0.10).

#### 3.3.4. Discuss HIV Care with HIV-Positive Alters

The participant characteristics associated with discussing HIV care with HIV positive network members were the age of the participant and gender: age was negatively correlated with discussing HIV care among HIV positive network members (*r* = −0.41, *P* < 0.01); and women (81%) were more likely to discuss HIV care with their HIV positive network members than men (56%) (*P* < 0.05). Length of time on ART and the number of isolated network members were both associated at a trend level of significance (*P* values <0.10). No network composition characteristics were associated with discussing HIV care with HIV-positive alters.

## 4. Discussion

This is one of the few studies to have explored the relationship between the social networks of PLHAs and their engagement in HIV prevention advocacy. We observed that all participants had discussed HIV and advocated for specific HIV protective behaviours (i.e., HIV testing, condom use, seeking HIV care) with at least one alter, and on average, participants had engaged in these prevention advocacy discussions with 50–70% of the members in their network. These findings imply that prevention advocacy may be a relatively common and natural behaviour of PLHA, particularly those in HIV care.

Our findings indicate that the proportion of alters who know the PLHAs HIV status may be the most influential social network characteristic with regard to the proportion of network members with whom HIV protective behaviours are discussed. This network characteristic was associated with the three advocacy behaviours that relate to HIV in general or HIV prevention—discussion of HIV, abstinence or condom use, and seeking an HIV test. The important role of the social network's knowledge of the PLHAs HIV status highlights the significance of HIV disclosure to HIV prevention advocacy. Our prior analysis of the social network data from this sample revealed that the participants had generally surrounded themselves with alters who they felt were supportive and trustworthy, and who were mostly peers and family members [22]. In the context of having friends and family who one can trust and rely on, it is not surprising that such individuals are comfortable disclosing their status within their network, and also discussing HIV and protective behaviours. Though not in the context of social network research, the importance of disclosure to prevention efforts is consistent with the findings of other studies [23, 24].

Our prior analysis revealed that the networks of our sample were generally dense and had high levels of interconnectedness, suggesting that information and attitudes such as messages encouraging HIV prevention, testing and care could rapidly travel through the network. In the analysis reported here, measures of network structure revealed that greater interconnectedness and less isolation and fragmentation of alters were at least marginally associated with prevention advocacy on the part of the respondent. The combination of these findings reveals the potential for PLHA to make a tremendous impact on HIV prevention in their families and communities.

With regard to the prevention advocacy behaviour of encouraging HIV-positive members to seek HIV care, the correlates of this behaviour were only with regard to the participant's individual characteristics. In particular, being younger or female was associated with discussing HIV care with HIV positive alters, compared to being older or male. A possible explanation for this gender difference is that women may be socialized to act as caregivers more so than men, which may contribute to women being more likely to encourage others in need to seek medical care.

The findings also highlight the role of ART and adherence in HIV prevention advocacy among PLHA. Being on ART and on ART for a longer time were marginally associated with discussing HIV in general and the need to seek HIV medical care, respectively, and ART adherence was significantly associated with discussing HIV with more network members. Perhaps people who are on ART and who adhere to treatment experience greater benefits from HIV care and thus have stronger beliefs in the value of HIV treatment and care. This in turn may motivate them more to discuss HIV and encourage others to seek treatment.

There are several limitations to be considered in interpreting these findings. The study was designed to be exploratory, not to generate population level parameter estimates or to be representative. We are not able to generalize to all PLHA in Uganda and the region because those in HIV care (who comprise all of our convenience sample) may be less stigmatized, more comfortable disclosing their HIV status, and have greater social support since they must have a “treatment supporter” within their social network to be eligible for ART—a common requirement for receipt of ART in much of sub-Saharan Africa. We elicited only 20 alters per respondent, which we expect to be sufficient to establish basic information about the networks of this population, but a larger number of alters may have allowed us to capture a more complete range of the members of these networks, particularly nonfamily network members such as coworkers. Therefore, our data speak more so to prevention advocacy within the relative inner circle of the social networks of PLHA. The cross-sectional nature of the study limits the ability to capture the effects of time on ART and in HIV care on the composition and structure of social networks and engagement in prevention advocacy. And finally, the small sample size limited our statistical power and it is likely that several of the marginal findings would have been significant otherwise. 

At a time when new innovative concepts for HIV prevention interventions are needed to make further inroads against the spread of the HIV epidemic, our study data suggest that PLHA have the potential to serve as a key part of the solution, not only in regards to reducing their own risk behaviour, but also as powerful agents for health behaviour change and HIV prevention among their families, friends and community. Social network-based interventions that facilitate and empower PLHA to strengthen their engagement in HIV prevention advocacy behaviours, which are already common and occurring naturally among many PLHA, could allow prevention messages to penetrate entire communities at a faster rate and more effectively than existing interventions that mostly target individuals. Comfort with HIV disclosure appears to play a key role in enabling PLHA to be comfortable engaging in prevention advocacy, indicating the importance of efforts to reduce both internalized HIV stigma and community-based HIV stigma and discrimination. Further research is now needed to build upon this initial preliminary data, to learn more about the benefits as well as potential risks associated with PLHA engaging in HIV prevention advocacy, and to develop and test interventions that enable PLHA to safely and effectively serve as agents for HIV prevention, thereby no longer being viewed as the source of the HIV problem, but the central force behind the solution to the HIV epidemic.

## Figures and Tables

**Table 1:** Correlates of engagement in prevention advocacy behaviours with proportion of network members (alters).

	Prevention advocacy behaviours			
	HIV in general	Abstinence/condom use	Seek HIV testing	Seek HIV care
Respondent Characteristics				

Age	*r* = 0.27_~_	*r* = 0.12	*r* = 0.08	*r* = −0.41**
Gender	*F* = 0.00	*F* = 0.02	*F* = 0.03	*F* = 4.77*
	Male = 60%	Male = 49%	Male = 48%	Male = 81%
	Female = 60%	Female = 48%	Female = 46%	Female = 56%
Time since HIV diagnosis	*r* = 0.44**	*r* = 0.31_~_	*r* = 0.31_~_	*r* = −0.02
On ART	*F* = 2.77_~_	*F* = 0.32	*F* = 0.85	*F* = 1.09
	No = 54%	No = 46%	No = 43%	No = 79%
	Yes = 67%	Yes = 50%	Yes = 52%	Yes = 67%
Time on ART (months)^1^	*F* = 1.44	*F* = 0.51	*F* = 0.58	*F* = 3.10_~_
	0 = 55%	0 = 47%	0 = 44%	0 = 80%
	6 = 60%	6 = 44%	6 = 45%	6 = 20%
	12 = 73%	12 = 55%	12 = 57%	12 = 48%
ART adherence	*r* = 0.55**	*r* = 0.31	*r* = 0.31	*r* = 0.12
Peer community support	*r* = 0.20	*r* = 0.16	*r* = 0.08	*r* = 0.20

Network composition				

% of alters who know respondent is HIV+	*r* = 0.60***	*r* = 0.35*	*r* = 0.41**	*r* = 0.06
% of alters who are HIV+	*r* = 0.29_~_	*r* = 0.23	*r* = 0.22	*r* = −0.03

Network structure				

Most popular contact	*r* = 0.35*	*r* = 0.19	*r* = 0.27_~_	*r* = 0.08
Number of isolates	*r* = −0.26	*r* = −0.22	*r* = −0.19	*r* = −0.28_~_
Network density	*r* = 0.31_~_	*r* = 0.22	*r* = 0.31_~_	*r* = −0.04

~*P* < 0.10; *P < 0.05; **P < 0.01; ***P < 0.001.

^1^0 = non-ART participants; 6 = participants on ART for 6 months or less; 12 = participants on ART for 12 months or more.
